# Professional athletes have extraordinary skills for rapidly learning complex and neutral dynamic visual scenes

**DOI:** 10.1038/srep01154

**Published:** 2013-01-31

**Authors:** Jocelyn Faubert

**Affiliations:** 1 NSERC-Essilor Industrial Research Chair, Visual Psychophysics and Perception Laboratory, School of Optometry, University of Montreal

## Abstract

Evidence suggests that an athlete's sports-related perceptual-cognitive expertise is a crucial element of top-level competitive sports^1^. When directly assessing whether such experience-related abilities correspond to fundamental and non-specific cognitive laboratory measures such as processing speed and attention, studies have shown moderate effects leading to the conclusion that their special abilities are context-specific^2^. We trained 308 observers on a complex dynamic visual scene task void of context and motor control requirements^3^ and demonstrate that professionals as a group dramatically differ from high-level amateur athletes, who dramatically differ from non-athlete university students in their capacity to learn such stimuli. This demonstrates that a distinguishing factor explaining the capacities of professional athletes is their ability to learn how to process complex dynamic visual scenes. This gives us an insight as to what is so special about the elite athletes' mental abilities, which allows them to express great prowess in action.

What makes elite athletes so special? Do brains of athletes anatomically and functionally differ from non-athletes and does this difference relate to performance level? A recent paper showed that high-level athletes have increased cortical thickness in a few areas of the brain and that this increased anatomical volume is correlated with the level of athletic training^4^. One of the areas identified in the athlete brain as different from controls was the superior temporal sulcus (STS), which plays a particular role in socially relevant stimuli^5^ and biological motion perception^6^. Biological motion perception involves the visual systems' capacity to recognize complex human movements when they are presented as a pattern of a few moving dots. This task is recognized as a critical and fundamental ability of social relevance^7^, and is a very strong dynamic cue that can be used for collision avoidance^8^ and anticipate opponents' movements in sports^9^,^10^. This is further supported by a recent study showing that athletes may be superior to non-athletes for processing socially realistic multitasking crowd scenes involving pedestrians crossing streets^11^. The superior abilities of high-level athletes for sports specific and socially realistic scenes both correspond to stimuli to which athletes have been extensively exposed throughout their lifespan. We are still lacking strong evidence that such abilities represent fundamental perceptual-cognitive abilities that would be expressed in laboratory measures void of social or contextual content^2^.

The 3-dimensional multiple-object-tracking speed threshold task (3D-MOT) was recently proposed as an optimal training procedure for isolating critical mental abilities when processing dynamic scenes such as when navigating in traffic or during sports activities^3^. The method relies on particular features suggested to be fundamental such as; 1) distributing attention among a number of moving targets among distractors, known in the literature as Multiple Object Tracking^12^,^13^, 2) a large visual field 3) speed thresholds, and 4) binocular 3-dimensional cues (3D) (i.e. stereoscopic vision). The rationale for using such conditions has been described in detail elsewhere^3^. We tested a total of 308 individuals separated into three distinct groups based on their performance levels in sports to determine whether the level of sports performance can distinguish the learning rate capacities for this complex and neutral visual scene task.

## Results

A total of 102 professional players (mean age = 23,8 ± 5,5 SD, median 22) from three different sports including 51 professional soccer players (English Premier League (EPL)), 21 professional ice hockey players (National Hockey League (NHL)) and 30 professional rugby players (French Top 14 Rugby League (Top14)). We also tested a total of 173 elite amateurs (mean age = 23,5 ± 5,8 SD, median 22) with 136 from the NCAA university sports program in the US and 37 from a European Olympic sport-training center. We have also tested 33 non-athlete university students (mean age = 23,8 ± 5,0 SD, median 22) from the Université de Montréal.

We have previously reported that, given identical conditions, top professional soccer, ice hockey or rugby teams generate very similar sensitivity profiles^3^. For this reason the professionals are presented as a single population group. Similarly, we obtained identical functions for our two amateur cohorts (NCAA and Olympic training center) studied here so again, we show the elite amateurs as one group.

**Figure 1:**
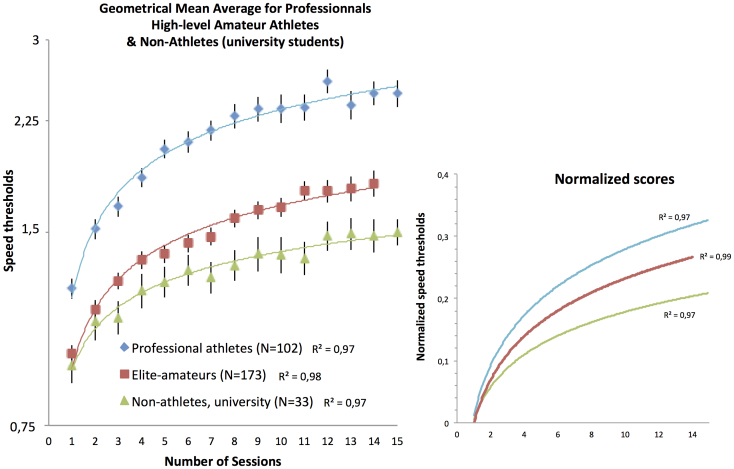
Geometrical 3D-MOT speed threshold means for 308 individuals on a log scale separated into professional, elite-amateur and non-athlete university students as a function of training sessions. The y values are arbitrary speed units. Only 14 sessions are shown for the amateurs because the protocol for the Olympic training center athletes was pre-set to terminate at 14 sessions. Error bars represent SEM.

Figure 1 shows the session-by-session geometrical mean graphs for the three groups with the session number on the x-axis and the 3D-MOT speed thresholds on a log y-axis. The fits shown are log regression functions and the R^2^ corresponds to the amount of variance explained by the fit. The data clearly show that the professional athlete group starts at higher speed values with a much steeper learning slope as a function of training session then the elite amateurs. In turn, the elite-amateur group starts at the same level as the non-athletes but the learning function rapidly distances itself from the one obtained for thenon-athlete university group. To emphasize the learning rate differences between the groups, the small graph on the right shows the normalized data (Log(sessions score) – Log(initial score)). One can see that the three learning rate functions are distinct regardless of the initial starting point scores.

## Discussion

The present results show a clear distinction between the level of athletic performance and corresponding fundamental mental capacities for learning an abstract and demanding dynamic scene task. How would this exceptional ability translate to specific real-life situations? For athletes, it is obviously related to their high levels of competitive sport performance. But what actions can we predict are enhanced by such a specialised ability for learning dynamic complex scenes? It would make logical sense that high-level athletes should be superior for achieving biological motion perception skills for instance. This is supported by the fact that cortical thickness of STS, an area known to process socially relevant cues and biological motion perception^5^, is greater and linked to training experience in athletes^4^. In other populations such as healthy older observers it has been shown that training with the 3D-MOT results in a direct subsequent transfer benefit to biological motion perception abilities at distances critical for collision avoidance^14^. The 3D-MOT speed task strongly engages several attention and mental skills that should carry over to other functions. To achieve high levels on this task one requires exquisite selective, dynamic, distributed and sustained attention skills for brief yet intense periods. Such abilities are certainly necessary when engaged in activities requiring the integration of simultaneous inputs such as when driving, crossing busy streets or when engaged in sporting activities. We have previously shown that the condition of testing can influence the learning curve^3^. This was demonstrated by the fact that if the professional players were standing as opposed sitting down for the initial consolidation training, the growth curve was reduced, which argues for shared resources. It remains to be determined whether this is specific to professional athletes or whether it can also be observed in other populations, as there clearly is something special about professional athletes. They appear to be able to hyper-focus for short periods of time resulting in extraordinary learning functions for the 3D-MOT task. We cannot determine here whether this superb ability to learn to process random and complex dynamic scenes has evolved by experience or stems from an innate predisposition. Prospective outcomes of athlete performance based on initial measures should prove very interesting in the future. The 3D-MOT method has been used to profile athletes for both the NHL and NFL combines where the best prospects for the entry draft are evaluated on a series of test batteries. It will be interesting to see whether these initial scores predict future performance outcomes. It is clear that individual performances on this task will be affected by many factors other than athletic skill including, sensory, physical, and psychological makeup so we should not expect a direct one to one relationship. It is clear that individual performances on this task will be affected by many factors other than athletic skill including, sensory, physical, and psychological makeup so we should not expect a direct one to one relationship. Nevertheless, our results do suggest that rapid learning in complex and unpredictable dynamic contexts is one of the critical components for elite performance.

In conclusion, we have demonstrated that professional athletes as a group have extraordinary skills for rapidly learning unpredictable, complex dynamic visual scenes that are void of any specific context. It is clear from these results that these remarkable mental processing and learning abilities should be acknowledged as critical elements for world-class performance in sport and potentially elite performance abilities in other dynamic contexts.

## Methods

**Figure 2:**
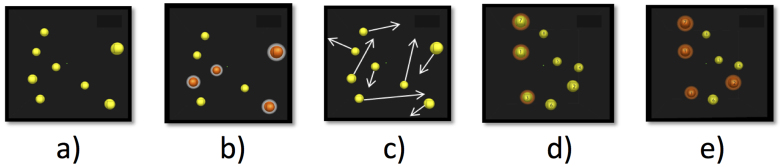
Five steps of the 3D-MOT task (a) presentation phase where 8 spheres are shown in a 3D volume space, (b) indexing phase where 4 spheres (targets) change colour (red) and are highlighted (hallo) for 1 second, (c) movement phase where the targets indexed in stage b return to their original form and colour and all spheres move for 8 seconds crisscrossing and bouncing off of each other and the virtual 3D volume cube walls that are not otherwise visible, (d) identification phase where the spheres come to a halt and the observer has to identify the 4 spheres originally indexed in phase (b). The spheres are individually tagged with a number so the observer can give the number corresponding to the original targets, and (e) feedback phase where the subject is given information on the correct targets.

The observers trained up to 15 sessions separated over a minimum of five different days using the NeuroTracker^TM^ CORE program distributed by CogniSens Athletics Inc., which is the commercial equivalent of the laboratory 3D-MOT speed threshold procedure that has been licenced by CogniSens Athletics Inc. from the Université de Montréal. Each session lasted around 8 minutes and the subjects were not allowed to train for more then three sessions in a given day. The basic 3-D MOT trial sequence is presented in Figure 2 and comprises of 5 steps (see legend).

The size of the 3D volume space was 46 degrees of visual angle at the level of the screen. After a single trial (Figure 2), if the subject got all 4 indexed spheres correct the speed went up for the next trial. If at least one sphere was missed the speed slowed down on the next trial (1 up 1 down staircase) so on and so forth until a threshold was achieved^3^. All subjects gave the answers verbally and an experimenter recorded the answers on a keyboard. This study was approved by the ethics board of the Université de Montréal.

## Author Contributions

J.F. wrote the manuscript text, did the analysis and prepared the figures.
